# A low-cost 2-D video system can accurately and reliably assess adaptive gait kinematics in healthy and low vision subjects

**DOI:** 10.1038/s41598-019-54913-5

**Published:** 2019-12-05

**Authors:** Tjerk Zult, Jonathan Allsop, Juan Tabernero, Shahina Pardhan

**Affiliations:** 1 Vision and Eye Research Institute, Faculty of Health, Education, Medicine, and Social Care, Anglia Ruskin University, Cambridge, United Kingdom; 2 Central Flying School, Royal Air Force College, College Cranwell, Sleaford, United Kingdom; 3 Departamento de Electromagnetismo y Electronica, Universidad de Murcia, Murcia, Spain

**Keywords:** Object vision, Ageing, Bone quality and biomechanics, Navigation

## Abstract

3-D gait analysis is the gold standard but many healthcare clinics and research institutes would benefit from a system that is inexpensive and simple but just as accurate. The present study examines whether a low-cost 2-D motion capture system can accurately and reliably assess adaptive gait kinematics in subjects with central vision loss, older controls, and younger controls. Subjects were requested to walk up and step over a 10 cm high obstacle that was positioned in the middle of a 4.5 m walkway. Four trials were simultaneously recorded with the Vicon motion capture system (3-D system) and a video camera that was positioned perpendicular to the obstacle (2-D system). The kinematic parameters (crossing height, crossing velocity, foot placement, single support time) were calculated offline. Strong Pearson’s correlations were found between the two systems for all parameters (average r = 0.944, all p < 0.001). Bland-Altman analysis showed that the agreement between the two systems was good in all three groups after correcting for systematic biases related to the 2-D marker positions. The test-retest reliability for both systems was high (average ICC = 0.959). These results show that a low-cost 2-D video system can reliably and accurately assess adaptive gait kinematics in healthy and low vision subjects.

## Introduction

The preferred approach for the kinematic assessment of gait is a three-dimensional (3-D) motion analysis system incorporating multiple cameras and active (light-emitting diodes), or passive (retroreflective) markers. The 3-D analysis of gait is highly accurate and reliable^1^ but the purchase of a 3-D motion analysis system is expensive and can be prohibitive for many, especially in developing countries. Many clinicians and researchers cannot afford such a system^2^, particularly in low-income countries. Clinicians therefore rely on qualitative/observational gait analysis to detect gait abnormalities and examine the effectiveness of an intervention. However, the outcomes of qualitative gait analysis are subjective and lack sufficient reliability^3–5^. Therefore a gait analysis systems that is cost effective and reliable would be a big advantage for clinical evaluations.

In the last 10 years, several gait analysis systems have been developed to perform quantitative gait analysis using a single video camera^6–10^. These low-cost gait assessment techniques provide valid kinematic data^6–8^ but the kinematic outcomes have been limited to joint angles and walking speed during walking gait. To date, there is no low-cost gait analysis system that has been shown to measure the kinematics of adaptive gait such as the negotiation of an obstacle.

The kinematic assessment of obstacle negotiation provides an insight in how well people can integrate information from sensory and motor systems common to many visually guided movements^11^. The spatiotemporal parameters that are often examined during obstacle negotiation are crossing height, crossing velocity, single support time during crossing, and foot placement before and after crossing the obstacle^12,13^. The obstacle crossing strategy changes with age as older adult tend to lift their feet higher and slower over the obstacle and place their feet closer to the obstacle before and after crossing^12,13^. These changes in obstacle crossing are likely mediated by an age-related decline in muscle strength, proprioception, vision, and cognitive function^12,14,15^.

The effect of vision impairment on obstacle negotiation and step ascent has also been widely studied^16–18^. Compared to age-matched controls, subjects with central vision loss due to age-related macular degeneration (AMD) lifted both feet higher and slower over the obstacle^17^ and exhibited a slower lead foot crossing velocity and swing time during step ascent^18^. Thus, the kinematic assessment of obstacle crossing and step ascent can help to detect changes in adaptive gait that are related to deficits in sensorimotor integration. Other examples include the influence of sensory substitution devices and echolocation on the navigation around obstacles^19–22^. However, all these aforementioned adaptive gait studies used an expensive 3-D motion analysis system to examine movement kinematics in healthy and low vision patients.

The present study aims to test a reliable and low-cost motion analysis system for the kinematic assessment of adaptive gait on subjects with normal vision and those with central field loss. Key spatiotemporal parameters for obstacle negotiation of a low-cost 2-D system (single video camera with a bull’s eye marker system) will be compared to the gold standard 3-D Vicon motion analysis system. The validity of the system will be determined for three population subgroups in which adaptive gait is commonly examined (i.e., healthy young adults, healthy older adults, and older adults with central field loss due to AMD).

## Results

### Participant characteristics

The participant characteristics can be found in Table 1. The results of the ANOVA show that the groups were significantly different in age, visual acuity, and contrast sensitivity (all p < 0.001). Post hoc testing revealed that AMD subjects were significantly older than older controls and younger controls (both p ≤ 0.042), and that older controls were older than younger controls (p < 0.001). Visual acuity and contrast sensitivity were significantly worse for participants with AMD than older and younger controls (both p < 0.001) and did not significantly differ between older and younger controls (p ≥ 0.391). However, the between-group differences in age are not a problem because the present study aims to examine kinematic differences between systems and not between groups.

**Table 1:** Group characteristics of the participants (mean ± SD).

	AMD subjects (n = 20)	Older controls (n = 13)	Younger controls (n = 14)
Age *(years)*	75 (7)	70 (4)*	27 (5)*^†^
**Gender**			
Male	10	6	7
Female	10	7	7
Mass *(kg)*	74 (13)	70 (11)	66 (14)
Height *(cm)*	170 (10)	168 (8)	170 (11)
Visual acuity *(logMAR)*	0.41 (0.38)	0.00 (0.12)*	−0.15 (0.09)*
Contrast sensitivity *(logCS)*	1.03 (0.43)	1.70 (0.07)*	1.77 (0.12)*

AMD, age-related macular degeneration; *significantly different compared to AMD participants (p < 0.05); ^†^significantly different compared to older controls (p < 0.05).

### Agreement between the 2-D and 3-D system

**Figure 1:**
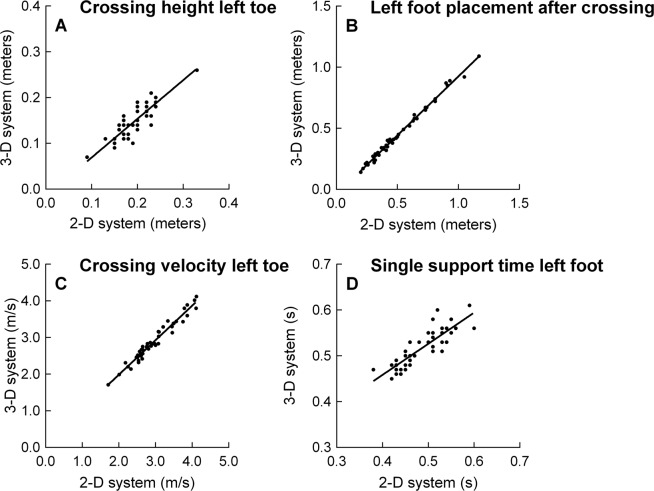
Pearson’s correlations between a representative set of movement kinematics measured with the 2-D and 3-D motion capture system. The Pearson’s correlations were strong to very strong for crossing height left toe (r = 0.873, p < 0.001), left foot placement after crossing (r = 0.996, p < 0.001), crossing velocity left toe (r = 0.979, p < 0.001), and single support time left foot (r = 0.851, p < 0.001). All subjects are included in the plots (n = 47).

Figure 1 shows a representative Pearson’s correlation for crossing height, foot placement, crossing velocity, and single support time when measured with the 2-D vs. 3-D motion capture system. An overview of all Pearson’s correlations can be found in Table 2. There was a strong to very strong positive correlation between the kinematic variables measured with the 2-D and 3-D motion capture system (r = 0.755 to 0.997, average r = 0.944, all p < 0.001).

**Table 2:** Pearson’s correlations (r) between the kinematic outcomes of the 2-D and 3-D motion capture system.

Variables	Total (n = 47)	AMD subjects (n = 20)	Older controls (n = 13)	Younger controls (n = 14)
Crossing height right toe	0.855	0.874	0.922	0.755
Crossing height left toe	0.873	0.909	0.810	0.841
Crossing height right heel	0.945	0.972	0.980	0.934
Crossing height left heel	0.973	0.987	0.950	0.974
Crossing velocity right toe	0.928	0.929	0.908	0.928
Crossing velocity left toe	0.979	0.984	0.984	0.971
Right foot placement before crossing	0.991	0.994	0.983	0.995
Left foot placement before crossing	0.991	0.984	0.997	0.994
Right foot placement after crossing	0.990	0.993	0.997	0.985
Left foot placement after crossing	0.996	0.998	0.994	0.993
Single support time right foot	0.961	0.908	0.987	0.955
Single support time left foot	0.851	0.872	0.906	0.834

Note: all correlations were significant (p < 0.001). AMD, age-related macular degeneration.

**Figure 2:**
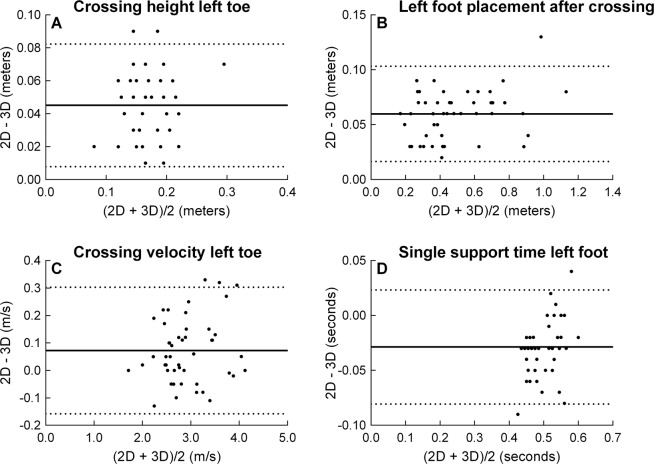
Bland-Altman plots that show the agreement between a representative set of movement kinematics measured with the 2-D and 3-D motion capture system. All subjects are included in the plots (n = 47). In each graph, the solid black line represents the mean bias and the dotted lines represent the 95% limits of agreement.

Figure 2 demonstrates a representative Bland-Altman plot for crossing height, foot placement, crossing velocity, and single support time. The data and results of the Bland-Altman analysis for all kinematic variables are shown in Table 3. The uncorrected crossing height variables were 0.04–0.05 m higher with the 2-D vs. 3-D system. After correction, the crossing height variables differed −0.03 to 0.01 m when analysed with the 2-D vs. 3-D system. The uncorrected foot placement variables were 0.05–0.06 m higher with the 2-D vs. 3-D system. After correction, the foot placement variables differed −0.01 to 0.03 m when analysed with the 2-D vs. 3-D system. Compared to the 3-D system, the crossing velocity measured with the 2-D system was 0.07 m/s faster for the left toe and 0.30 m/s faster for the right toe. After correction, the crossing velocity of the right toe differed −0.01 m/s when analysed with the 2-D vs. 3-D system. No correction was applied to the crossing velocity of the left toe. The single support times were 0.00–0.03 s shorter with the 2-D vs. 3-D system.

**Table 3:** Bland-Altman analysis between the kinematic outcomes of the 2-D and 3-D motion capture system.

Variables	Groups		2-D uncorrected			2-D corrected^1,2^		
		3-D system (mean ± SD)	2-D system (mean ± SD)	2-D – 3-D (mean ± SD)	2-D – 3-D (% of 3-D)	2-D system (mean ± SD)	2-D – 3-D (mean ± SD	2-D – 3-D (% of 3-D)
Crossing height right toe (m)	Total (n = 47)	0.14 (0.04)	0.19 (0.05)	0.05 (0.03)	35%	0.14 (0.05)	0.00 (0.025)	3%
	AMD subjects (n = 20)	0.15 (0.05)	0.20 (0.05)	0.06 (0.03)*	40%	0.16 (0.05)	0.01 (0.03)	9%
	Older controls (n = 13)	0.14 (0.05)	0.19 (0.05)	0.05 (0.02)	34%	0.15 (0.05)	0.00 (0.021)	2%
	Younger controls (n = 14)	0.13 (0.04)	0.17 (0.04)	0.04 (0.02)	27%	0.13 (0.04)	−0.01 (0.02)	−11%
Crossing height left toe (m)	Total (n = 47)	0.15 (0.04)	0.19 (0.04)	0.04 (0.02)	31%	0.15 (0.04)	0.00 (0.02)	0%
	AMD subjects (n = 20)	0.15 (0.04)	0.19 (0.05)	0.04 (0.02)	29%	0.15 (0.05)	0.00 (0.02)	−1%
	Older controls (n = 13)	0.14 (0.04)	0.19 (0.03)	0.05 (0.02)	34%	0.15 (0.03)	0.00 (0.02)	2%
	Younger controls (n = 14)	0.14 (0.03)	0.19 (0.03)	0.05 (0.02)	31%	0.14 (0.03)	0.00 (0.02)	−1%
Crossing height right heel (m)	Total (n = 47)	0.25 (0.09)	0.28 (0.09)	0.04 (0.03)	16%	0.24 (0.09)	−0.01 (0.03)	−2%
	AMD subjects (n = 20)	0.24 (0.09)	0.30 (0.11)	0.05 (0.03)*	22%	0.25 (0.11)	0.01 (0.03)	3%
	Older controls (n = 13)	0.20 (0.07)	0.25 (0.08)	0.04 (0.02)*	22%	0.21 (0.08)	0.00 (0.02)	1%
	Younger controls (n = 14)	0.29 (0.07)	0.30 (0.07)	0.02 (0.02)	5%	0.26 (0.07)	−0.03 (0.02)	−11%
Crossing height left heel (m)	Total (n = 47)	0.21 (0.09)	0.26 (0.08)	0.04 (0.02)	22%	0.21 (0.08)	0.00 (0.02)	0%
	AMD subjects (n = 20)	0.20 (0.10)	0.26 (0.09)	0.05 (0.02)^†^	25%	0.21 (0.09)	0.01 (0.02)	3%
	Older controls (n = 13)	0.23 (0.07)	0.26 (0.07)	0.03 (0.02)	15%	0.22 (0.07)	−0.01 (0.02)	−5%
	Younger controls (n = 14)	0.20 (0.08)	0.25 (0.07)	0.05 (0.02)	23%	0.20 (0.07)	0.00 (0.02)	1%
Crossing velocity right toe (m/s)	Total (n = 47)	2.81 (0.54)	3.11 (0.56)	0.30 (0.21)	11%	2.80 (0.50)	−0.01 (0.20)	0%
	AMD subjects (n = 20)	2.58 (0.51)	2.93 (0.55)	0.35 (0.20)	14%	2.63 (0.50)	0.06 (0.19)	2%
	Older controls (n = 13)	3.00 (0.36)	3.31 (0.38)	0.31 (0.16)	10%	2.98 (0.35)	−0.03 (0.15)	−1%
	Younger controls (n = 14)	2.98 (0.60)	3.20 (0.65)	0.22 (0.24)	7%	2.88 (0.59)	−0.10 (0.23)	−3%
Crossing velocity left toe (m/s)	Total (n = 47)	2.88 (0.55)	2.96 (0.57)	0.07 (0.12)	3%	N/A	N/A	N/A
	AMD subjects (n = 20)	2.70 (0.48)	2.76 (0.54)	0.06 (0.11)	2%	N/A	N/A	N/A
	Older controls (n = 13)	2.92 (0.59)	2.98 (0.54)	0.07 (0.11)	2%	N/A	N/A	N/A
	Younger controls (n = 14)	3.12 (0.54)	3.21 (0.56)	0.10 (0.13)	3%	N/A	N/A	N/A
Right foot placement before crossing (m)	Total (n = 47)	0.48 (0.25)	0.54 (0.26)	0.06 (0.04)	13%	0.50 (0.26)	0.02 (0.04)	3%
	AMD subjects (n = 20)	0.46 (0.28)	0.53 (0.29)	0.07 (0.03)	14%	0.48 (0.29)	0.02 (0.03)	4%
	Older controls (n = 13)	0.56 (0.22)	0.64 (0.23)	0.08 (0.04)*	14%	0.59 (0.23)	0.03 (0.04)	6%
	Younger controls (n = 14)	0.44 (0.23)	0.48 (0.24)	0.04 (0.03)	9%	0.44 (0.24)	0.01 (0.03)	−1%
Left foot placement before crossing (m)	Total (n = 47)	0.60 (0.23)	0.64 (0.24)	0.05 (0.03)	8%	0.60 (0.24)	0.00 (0.03)	0%
	AMD subjects (n = 20)	0.56 (0.21)	0.61 (0.22)	0.04 (0.04)	8%	0.56 (0.22)	0.00 (0.04)	0%
	Older controls (n = 13)	0.55 (0.24)	0.60 (0.25)	0.05 (0.02)	9%	0.55 (0.25)	0.01 (0.02)	1%
	Younger controls (n = 14)	0.69 (0.23)	0.74 (0.24)	0.06 (0.02)	8%	0.69 (0.24)	−0.01 (0.02)	1%
Right foot placement after crossing (m)	Total (n = 47)	0.57 (0.25)	0.63 (0.26)	0.06 (0.04)	11%	0.59 (0.26)	0.02 (0.04)	3%
	AMD subjects (n = 20)	0.53 (0.25)	0.60 (0.28)	0.07 (0.04)	14%	0.55 (0.28)	0.03 (0.04)	5%
	Older controls (n = 13)	0.52 (0.25)	0.57 (0.25)	0.06 (0.02)	11%	0.53 (0.25)	0.01 (0.02)	2%
	Younger controls (n = 14)	0.69 (0.23)	0.74 (0.23)	0.05 (0.04)	8%	0.69 (0.23)	0.01 (0.04)	1%
Left foot placement after crossing (m)	Total (n = 47)	0.46 (0.22)	0.52 (0.23)	0.06 (0.02)	13%	0.48 (0.23)	0.01 (0.02)	3%
	AMD subjects (n = 20)	0.41 (0.26)	0.47 (0.27)	0.06 (0.02)	15%	0.43 (0.27)	0.02 (0.02)	4%
	Older controls (n = 13)	0.56 (0.21)	0.61 (0.21)	0.05 (0.02)	9%	0.56 (0.21)	0.01 (0.02)	1%
	Younger controls (n = 14)	0.45 (0.17)	0.52 (0.17)	0.06 (0.02)	14%	0.47 (0.17)	0.02 (0.02)	4%
Single support time right foot (s)	Total (n = 47)	0.54 (0.06)	0.54 (0.06)	0.00 (0.02)	1%	N/A	N/A	N/A
	AMD subjects (n = 20)	0.56 (0.04)	0.56 (0.05)	0.01 (0.02)	1%	N/A	N/A	N/A
	Older controls (n = 13)	0.53 (0.09)	0.53 (0.09)	0.00 (0.02)	0%	N/A	N/A	N/A
	Younger controls (n = 14)	0.53 (0.05)	0.53 (0.05)	0.01 (0.02)	1%	N/A	N/A	N/A
Single support time left foot (s)	Total (n = 47)	0.52 (0.04)	0.49 (0.05)	−0.03 (0.03)	−6%	N/A	N/A	N/A
	AMD subjects (n = 20)	0.53 (0.04)	0.50 (0.05)	−0.03 (0.03)	−6%	N/A	N/A	N/A
	Older controls (n = 13)	0.51 (0.04)	0.49 (0.06)	−0.02 (0.02)	−4%	N/A	N/A	N/A
	Younger controls (n = 14)	0.52 (0.04)	0.48 (0.04)	−0.03 (0.02)	−6%	N/A	N/A	N/A

Note: between-group comparisons have only been performed for the difference scores between the 2-D and 3-D system (i.e., 2D – 3D, fifth column). 1, a correction factor of −0.045 m was applied to correct the systematic bias between the 2-D and 3-D system for all the crossing height and foot placement variables; 2, the following correction was applied to the 2D crossing velocity of the right toe to correct the systematic bias between the 2-D and 3-D system: 2D crossing velocity right toe – (0.10 * 2D crossing velocity right toe); AMD, age-related macular degeneration; N/A: not applicable; *significantly different compared to younger controls (p < 0.05); ^†^significantly different compared to older controls (p < 0.05).

The results of the mixed ANOVA with uncorrected 2-D data show that the bias scores (i.e., difference in score between the 2-D and 3-D measurement system) were significantly different between the groups for crossing height (right toe, right heel, and left heel) and right foot placement before crossing (all p ≤ 0.048). Post hoc analysis revealed that the bias for the crossing height of the right toe was 0.02 m less in younger controls than AMD subjects (p = 0.043, d = −0.85, 95% CI from −0.04 to 0.00). The bias for the crossing height of the right heel was 0.03 m less in younger controls compared to older controls (p = 0.010, d = −1.35, 95% CI from −0.05 to −0.01) and AMD participants (p < 0.001, d = −1.32, 95% CI from −0.06 to −0.01). The bias for the crossing height of the left heel was 0.02 m less in older controls than AMD subjects (p = 0.019, d = −1.07, 95% CI from −0.04 to 0.00). The bias for right foot placement before crossing was 0.04 m less in younger than older controls (p = 0.024, d = −1.01, 95% CI from −0.07 to 0.00). Post hoc analysis did not show any other significant differences between groups (all p ≥ 0.069).

### Test-retest reliability

Table 4 shows that both systems show good test-retest reliability for each kinematic variable. The intraclass correlation coefficients (ICCs) indicate a moderate to excellent test-retest reliability for the 2-D system (ICC = 0.624 to 0.997, average ICC = 0.944, all p < 0.001) and a good to excellent test-retest reliability for the 3-D system (ICC = 0.745 to 0.996, average ICC = 0.954, p < 0.001). The bias scores (i.e., difference score between the test and retest) were between −0.004 and 0.015 m for crossing height, between −0.019 and 0.030 m for foot placement, between −0.140 and 0.123 m/s for crossing velocity, and between −0.007 and 0.019 s for single support time. The results of the mixed ANOVA show that the bias scores were not significantly different between groups (all p ≥ 0.076) and systems (all p ≥ 0.174) and there were no significant interactions (all p ≥ 0.117).

**Table 4:** Test-retest reliability of the kinematic outcomes of the 2-D and 3-D motion capture system.

Variables	Groups	2-D system			3-D system		
		test – retest (mean ± SD)	test – retest (% of the mean)	ICC	test – retest (mean ± SD)	test – retest (% of the mean)	ICC
Crossing height right toe (m)	Total (n = 47)	−0.001 (0.028)	0%	0.933	0.000 (0.023)	0%	0.946
	AMD subjects (n = 20)	−0.004 (0.026)	−2%	0.940	−0.003 (0.019)	−2%	0.957
	Older controls (n = 13)	0.005 (0.028)	3%	0.945	0.007 (0.019)	5%	0.970
	Younger controls (n = 14)	−0.002 (0.031)	−1%	0.890	−0.002 (0.029)	−1%	0.901
Crossing height left toe (m)	Total (n = 47)	0.006 (0.033)	3%	0.924	0.003 (0.025)	2%	0.924
	AMD subjects (n = 20)	0.002 (0.034)	1%	0.936	0.003 (0.020)	2%	0.967
	Older controls (n = 13)	0.015 (0.024)	8%	0.944	0.009 (0.019)	6%	0.954
	Younger controls (n = 14)	0.001 (0.038)	1%	0.845	0.000 (0.036)	0%	0.745
Crossing height right heel (m)	Total (n = 47)	0.000 (0.033)	0%	0.981	0.004 (0.026)	2%	0.988
	AMD subjects (n = 20)	−0.002 (0.033)	−1%	0.983	0.003 (0.023)	1%	0.990
	Older controls (n = 13)	0.009 (0.028)	4%	0.983	0.012 (0.022)	6%	0.988
	Younger controls (n = 14)	−0.004 (0.037)	−1%	0.967	−0.002 (0.034)	0%	0.975
Crossing height left heel (m)	Total (n = 47)	0.005 (0.033)	2%	0.971	0.002 (0.027)	1%	0.986
	AMD subjects (n = 20)	0.002 (0.031)	1%	0.980	0.005 (0.018)	3%	0.994
	Older controls (n = 13)	0.012 (0.020)	5%	0.986	−0.001 (0.019)	0%	0.994
	Younger controls (n = 14)	0.003 (0.043)	1%	0.924	0.001 (0.043)	0%	0.953
Crossing velocity right toe (m/s)	Total (n = 47)	−0.047 (0.267)	−2%	0.953	−0.038 (0.284)	−1%	0.948
	AMD subjects (n = 20)	−0.009 (0.299)	0%	0.945	0.014 (0.238)	1%	0.963
	Older controls (n = 13)	−0.005 (0.233)	0%	0.945	−0.007 (0.289)	0%	0.889
	Younger controls (n = 14)	−0.139 (0.242)	−4%	0.960	−0.140 (0.330)	−5%	0.939
Crossing velocity left toe (m/s)	Total (n = 47)	0.000 (0.348)	0%	0.924	−0.018 (0.250)	−1%	0.959
	AMD subjects (n = 20)	−0.065 (0.276)	−2%	0.936	−0.029 (0.191)	−1%	0.965
	Older controls (n = 13)	0.123 (0.313)	4%	0.944	0.031 (0.293)	1%	0.963
	Younger controls (n = 14)	−0.020 (0.451)	−1%	0.845	−0.046 (0.292)	−1%	0.924
Right foot placement before crossing (m)	Total (n = 47)	−0.008 (0.076)	2%	0.989	−0.011 (0.060)	1%	0.989
	AMD subjects (n = 20)	−0.019 (0.072)	4%	0.991	−0.007 (0.065)	3%	0.993
	Older controls (n = 13)	−0.008 (0.062)	1%	0.994	0.006 (0.100)	1%	0.992
	Younger controls (n = 14)	0.008 (0.095)	−2%	0.973	−0.005 (0.074)	−2%	0.967
Left foot placement before crossing (m)	Total (n = 47)	−0.008 (0.119)	1%	0.970	−0.007 (0.093)	1%	0.981
	AMD subjects (n = 20)	−0.019 (0.103)	1%	0.967	−0.028 (0.089)	5%	0.973
	Older controls (n = 13)	−0.016 (0.080)	−3%	0.990	−0.010 (0.069)	2%	0.993
	Younger controls (n = 14)	0.015 (0.168)	8%	0.929	0.026 (0.113)	−3%	0.965
Right foot placement after crossing (m)	Total (n = 47)	−0.001 (0.081)	0%	0.987	0.006 (0.064)	1%	0.991
	AMD subjects (n = 20)	0.004 (0.085)	1%	0.985	0.016 (0.062)	3%	0.989
	Older controls (n = 13)	−0.001 (0.042)	0%	0.997	−0.002 (0.048)	0%	0.996
	Younger controls (n = 14)	−0.009 (0.104)	−1%	0.966	−0.002 (0.081)	0%	0.979
Left foot placement after crossing (m)	Total (n = 47)	0.007 (0.062)	1%	0.991	0.001 (0.056)	0%	0.993
	AMD subjects (n = 20)	0.007 (0.045)	1%	0.996	0.001 (0.041)	0%	0.996
	Older controls (n = 13)	−0.018 (0.088)	−3%	0.985	−0.022 (0.080)	−4%	0.987
	Younger controls (n = 14)	0.030 (0.049)	8%	0.988	0.022 (0.043)	7%	0.991
Single support time right foot (s)	Total (n = 47)	0.006 (0.030)	1%	0.939	0.001 (0.034)	1%	0.887
	AMD subjects (n = 20)	−0.001 (0.028)	3%	0.957	0.003 (0.035)	3%	0.904
	Older controls (n = 13)	0.018 (0.029)	−1%	0.944	0.008 (0.026)	−1%	0.934
	Younger controls (n = 14)	0.002 (0.033)	−1%	0.876	−0.007 (0.039)	−1%	0.790
Single support time left foot (s)	Total (n = 47)	0.005 (0.038)	1%	0.927	0.004 (0.035)	0%	0.931
	AMD subjects (n = 20)	0.019 (0.038)	0%	0.917	0.017 (0.032)	0%	0.925
	Older controls (n = 13)	−0.006 (0.034)	4%	0.935	−0.005 (0.029)	1%	0.951
	Younger controls (n = 14)	−0.004 (0.038)	0%	0.920	−0.006 (0.039)	−1%	0.911

ICC: intraclass correlation coefficient.

Note: no significant group effects (all p ≥ 0.076), system effects (all p ≥ 0.174) and interactions (all p ≥ 0.117) were observed. AMD, age-related macular degeneration.

## Discussion

The present study examined the validity of an inexpensive video system designed for the 2-D analysis of adaptive gait. The results showed that important spatiotemporal parameters of adaptive gait can be determined accurately and reliably with the low-cost 2-D analysis in healthy subjects and subjects with central vision loss.

Pearson’s correlation coefficients between the spatiotemporal outcomes of the 2-D and 3-D analysis were high to very high which indicate a strong relationship between the outcomes of the two systems. Our results support the findings of a previous study that showed strong correlations between the two systems for lower extremity joint angles during normal gait^8^. Bland-Altman analysis showed that the agreement between the 2-D and 3-D system was good for determining the feet’s single support times. To illustrate, single support times were only −6 to 1% different between the 2-D and 3-D analysis. The agreement between the 2-D and 3-D system was not as good for crossing height, foot placement, and crossing velocity but can be improved when the 2-D data is corrected for systematic biases.

Without correcting the 2-D data, the 2-D compared to the 3-D analysis resulted in a 2 to 6 cm (15 to 40%) higher crossing height and a 4 to 8 cm (8 to 15%) increase in foot placement away from the obstacle. The high discrepancy between the two systems is caused by the size and placement of the bull’s eye markers relative to the reflective markers of the 3-D system. The marker size for the 2-D system is 4.5 cm compared to 1.4 cm for the 3-D system- making the 3-D more sensitive than 2-D system. It was not possible to decrease the size of the bull’s eye markers as a smaller marker size could not be tracked with the Kinovea software. The size of the bull’s eye markers also made it difficult to place them at the same position as the 3-D markers. The 3-D toe marker was deliberately placed further forward on the shoe than the bull’s eye marker and the 3-D heel marker was deliberately placed further backward on the shoe than the bull’s eye marker. Altogether, both the size and placement of the bull’s eye markers caused a positive bias in crossing height and foot placement compared to the 3-D markers. However, this bias is consistent across trials and can be corrected for.

The difference in crossing velocity measured with the 2-D vs. 3-D system was 0.06 to 0.07 m/s (2 to 3%) for the left toe and 0.22 to 0.35 m/s (7 to 14%) for the crossing velocity of the right toe. The discrepancy in bias between the velocity estimate of the left and right toe is likely to be caused by a difference in bull’s eye marker placement for the left and right feet. In particular, the left toe marker of both systems was placed on the medial side of the shoe while the bull’s eye marker of the right toe was placed on the opposite shoe side (i.e., lateral) compared to the reflective marker of the 3-D system (i.e., medial). Placement of the toe’s bull’s eye marker on the medial side of the right shoe will therefore reduce the velocity error but the right shoe will block the camera’s view of the bull’s eye marker. Adding a second camera on the left side of the subject should solve this but it need another camera and would double the costs of the 2-D motion capture system. Alternatively, the velocity of the right toe’s bull’s eye marker can be corrected with a formula that takes into account the bias between the 2-D and 3-D system. Applying this formula reduces the difference in crossing velocity between the 2-D and 3-D system to −0.10 to 0.06 m/s (−3 to 2%) for the right toe, which is a similar bias as the left toe. Therefore, the 2-D system can be used to accurately measure the crossing velocity of the right and left toe when correcting the systematic bias for the right toe.

The amount of bias differed between groups for crossing height (right toe, right heel, left heel) and right foot placement before crossing. The bias between groups differed 2 to 3 cm for crossing height and 4 cm for foot placement. These group differences are too small to be of clinical significance. These small differences are likely caused by the aforementioned limitations of the bull’s eye marker system. In addition, the shape of the shoes (especially shoes with round edges) might have contributed to the group differences. The surface to stick the bull’s eye marker on is smaller for shoes with a round front and heel. Therefore, markers had to be placed further inwards (i.e., on the straighter edge of the shoes) when the shoes were round so that the marker did not fall off during walking. This is a limitation that needs to be taken into account in future studies.

Test-retest reliability was excellent for the 2-D and 3-D system and was not significantly different between systems and groups. The difference between the test and retest was −4 to 8% for the 2-D system and −5 to 7% for the gold standard 3-D system. In both systems, the lowest test-retest reliability was obtained for the crossing height variables. Irrespective of the used system, the difference between the test and retest was −2 to 5% for AMD subjects, −4 to 8% for older controls, and −5 to 8% for younger controls. Our data show that the test-retest reliability of the 2-D system is comparable to the gold standard 3-D system and that movement kinematics vary from trial to trial when negotiating an obstacle.

In conclusion, the 2-D video camera system offers an accurate and reliable alternative for the 3-D analysis of obstacle negotiation in healthy subjects and those with central vision loss. The 2-D system is inexpensive, easy to set up and the software required for the tracking of the markers is freely available. It shows good potential for researchers and clinicians to reliably assess adaptive gait using a cheaper 2-D system rather than relying on a subjective analysis to facilitate therapeutic outcomes and adaptive gait research in developing countries.

## Methods

### Participants

A total of 47 participants volunteered to take part in the study. Twenty of them were diagnosed with AMD, 13 were older adults with normal or corrected-to-normal vision, and 14 were young adults with normal or corrected-to-normal vision. The participants with AMD were recruited via a local support group and via letters that were sent out by the Macular Society. The older and younger adults with normal vision were recruited via online advertisement and word of mouth. To be included, participants had to be able to walk without a walking aid, AMD participants and older adults with normal vision had to be aged ≥ 60 years, and young adults with normal vision had to be aged 18–35 years. Exclusion criteria were: cognitive impairment, severe neurological or musculoskeletal problems, usage of medication that causes dizziness, and eye disorders or any other ocular pathology affecting eye sight (except AMD). The subjects’ health was evaluated through a self-report questionnaire and cognitive function was assessed with the Mini Mental State Examination. Subjects were classified as cognitively impaired when they scored below the population-based norm^23^. Two test were performed to examine eye sight. Binocular visual acuity was measured using the Bailey-Lovie logMAR chart at a working distance of 4 m using a letter-by-letter scoring system (0.02 logMAR)^24^. The distance was shortened when a participant was not able to read the largest size letters at 4 m distance and the score was adjusted accordingly. Binocular contrast sensitivity was examined with the Pelli-Robson chart at 1 m distance and scored per group of three letters (0.15 log units) of which two letters had to be correct^25^. A self-report questionnaire was used to assess the health of all participants. All AMD subjects were diagnosed with AMD in both eyes (dry or wet) by an ophthalmologist. The group characteristics are shown in Table 1.

### Experimental setup

**Figure 3:**
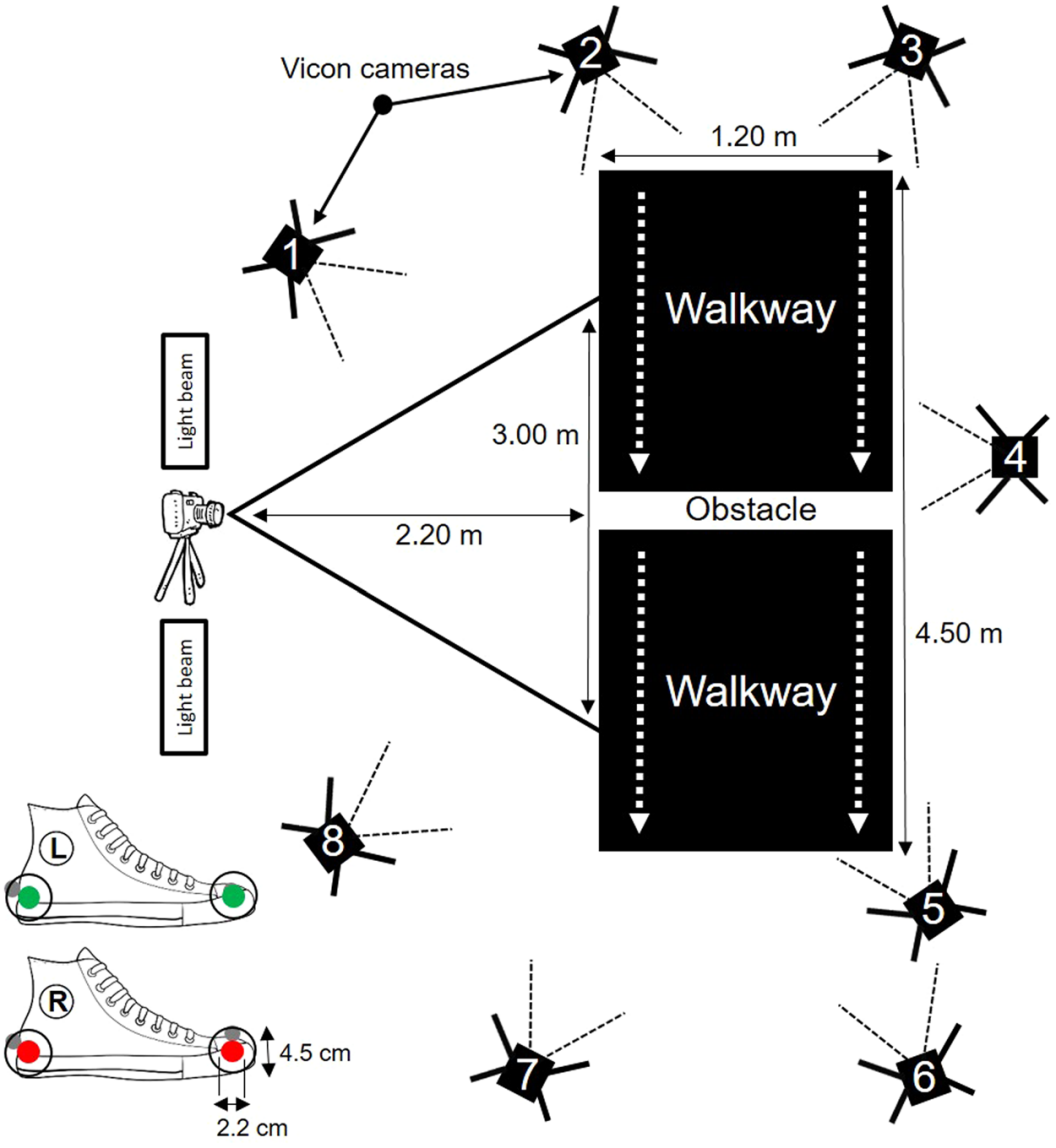
Schematic representation of the experimental setup. The grey markers on the shoes represent the reflective markers of the 3-D motion capture system. The markers of the 2-D motion capture system are red (right shoe) and green (left shoe). Note, the 3-D marker on the left toe is obstructed by the green 2-D marker.

Figure 3 shows a schematic representation of the experimental setup. The walkway was a black rubber mat that was positioned in the middle of a research lab. A 10 cm high obstacle was placed in the middle of the walkway and was made from light brown medium-density fibreboard and was 1.8 cm thick and 62 cm long^17^. The height of the obstacle reflected the typical step height encountered in daily life^17^. Participants were instructed to step over the obstacle while walking at their comfortable walking speed. The start position was at the beginning of the mat and the trial was finished when the participants reached the end of the mat. Participants performed the task with comfortable walking shoes. The walkway was surrounded by eight 3-D motion capture cameras (Vicon, Oxford Metrics Ltd). For the 2-D motion capture system, a camera with a high speed video recording mode (Lumix DMC-FZ200, Panasonic, Japan) was mounted on a 10 cm high tripod and positioned orthogonal to the obstacle. The camera was positioned on a 10 cm high tripod to match the camera height with the obstacle height. The distance between the camera and the obstacle was set to capture at least the foot placement of the left and right foot before and after crossing the obstacle. A light beam (Fly Series Flood Light, HC-GTG22–50W, Gosun, China) was positioned on each side of the photo camera to provide the recordings from sufficient lightning. Each participant performed four trials of which the last three trials were analysed. The experimental procedures were approved by the Research Ethics Committee of the Anglia Ruskin University and were in accordance with the Declaration of Helsinki. All participants provided written informed consent to the experimental procedures.

### 3-D motion capture

The 3-D motions were captured at a sampling frequency of 100 Hz using an eight camera Vicon Bonita system (Vicon, Oxford Metrics Ltd). A reflective marker (14 mm size) was placed on the first metatarsal head and the posterior part of the calcaneus of both the right and left shoe. The marker trajectories were tracked in Vicon Nexus 1.8.5 before the data was exported to Visual 3-D (C-Motion Inc., Rockville, MD., USA) for further analysis. In Visual 3-D, the kinematic data were filtered with a fourth-order low-pass Butterworth filter at 7 Hz and adaptive gait parameters were calculated using a custom build pipeline in Visual 3-D. A more detailed explanation of the adaptive gait parameters can be found in the section ‘kinematic parameters’.

### 2-D motion capture

The 2-D motions were captured in high speed video recording mode (100 Hz sampling rate) using a photo camera (Lumix DMC-FZ200, Panasonic, Japan). Videos of adaptive gait were recorded in the sagittal plane. Two bull’s eye markers were placed on each shoe. The internal and external dimensions of the makers are shown in Fig. 3 and were identical to previous work^8^. The dimensions of the bull’s eye markers were larger than the Vicon markers to improve track ability. Due to the unidimensional shape of the bull’s eye markers and the position of the photo camera, the markers on the right and left shoe were placed on the lateral and medial side of the shoe respectively, as illustrated in Fig. 3. In more detail, the right heel marker was placed at the lateral aspect of the heel, the right toe marker was placed at the lateral side of the fifth metatarsal head, the left heel marker was placed at the medial aspect of the heel, and the left toe marker was placed at the medial side of the first metatarsal head. Marker placement was in accordance with previous literature^8^.

**Figure 4:**
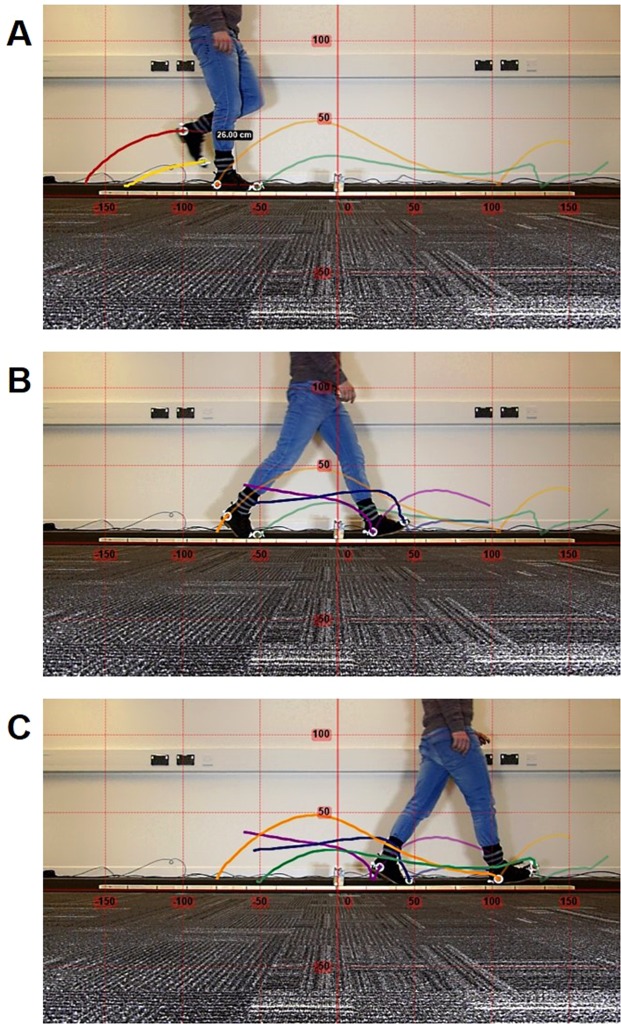
Marker trajectories in Kinovea during different phases of obstacle negotiation. The different colours represent the trajectories of different markers. Note that the marker trajectories for the left foot have a different colour before (**A**) compared to after (**B**, **C**) the markers were obstructed by the right leg.

The recorded videos were uploaded into Kinovea (version 0.8.15, available for download at: http://www.kinovea.org). In Kinovea, the internal dimension of each bull’s eye marker was tracked using the option ‘track path’ (see Fig. 4). Tracking of the left toe and left heel marker was lost when the left foot moved behind the right foot (see Fig. 4A for an illustration). Therefore, the path of these two markers was tracked separately before and after the occurrence of the marker obstruction and the two paths of each marker were merged for analysis. A coordinate system was placed on top of the marker paths with the origin at the point where the base of the obstacle contacted the ground (see Fig. 4). The coordinate system was calibrated by entering the actual distance between the heel and toe marker, as illustrated in Fig. 4A for the right foot. The initial idea was to use the ruler on the floor for calibration (see Fig. 4) but the position of the ruler is closer to the camera than the feet which caused a distortion effect (i.e., objects close to the lens appear larger relative to more distant objects). The effect of distortion was evident during piloting and therefore the calibration for the actual experiment was performed using the marker distance. Note that the calibration was performed separately for the right and left foot as the right foot was positioned closer to the camera than the left foot. The trajectories were exported to simple text and analysed using a custom build Mathematica script (Wolfram Research, Inc., Mathematica, Version 10, Champaign, Illinois). The exported data did not require filtering as the data showed a smooth and natural behaviour. Details about the calculation of the adaptive gait parameters can be found in the section ‘kinematic parameters’.

### Kinematic parameters

Various kinematic variables were examined, which have previously been identified as important in measuring obstacle negotiation^17,26,27^:

1. Vertical clearance height of the toe and heel at the point of crossing the obstacle.
2. Horizontal crossing velocity of the toe at the point of obstacle crossing.
3. Foot placement before crossing the obstacle – horizontal distance between the toe and the obstacle.
4. Foot placement after crossing the obstacle – horizontal distance between the heel and the obstacle.
5. Single support time of the left foot – swing time of the left foot during obstacle crossing when only the right foot is in contact with the ground.
6. Single support time of the right foot – swing time of the right foot during obstacle crossing when only the left foot is in contact with the ground.

The assessment of single support times required the calculation of heel strike and toe-off. For the 3-D analysis, heel strike was defined as the instant where the resultant velocity of the heel marker decreased below 0.6 m/s for ten consecutive frames. Heel strike for the 2-D analysis was defined as the instant where the vertical position of the heel marker decreased below 1.5 cm. Toe-off for the 3-D analysis was defined as the instant where the resultant velocity of the toe maker increased more 0.9 m/s for ten consecutive frames. For the 2-D analysis, toe off was defined as the instant where the vertical position of the toe marker increased above 1.5 cm. The threshold values for the 2-D and 3-D marker trajectories were determined by visual inspection of the kinematic data.

### Statistical analysis

The statistical computations were performed with SPSS (SPSS statistics 24.0, IBM, USA). Data in text and figures are expressed as mean ± SD. All demographic data were subjected to a mixed ANOVA to determine between-group differences, except that a Chi-square test was used to determine between-group differences in gender. The average of each kinematic parameter was calculated across the last three trials and used in the analysis. Pearson’s correlation and Bland-Altman analysis^28^ were performed to assess the agreement in kinematic outcomes between the 2-D vs. 3-D motion capture system. The 2-D data were compared with the 3-D data in uncorrected and corrected form for the crossing velocity of the right toe and all crossing height and foot placement variables. A correction factor of −0.045 m was applied to all crossing height and foot placement variables as this was calculated to be the systematic bias between the 2-D and 3-D system for those variables. The following formula was applied to correct the systematic bias between the 2-D and 3-D system for the crossing velocity of the right toe: 2D crossing velocity right toe – (0.10 * 2D crossing velocity of the right toe). A mixed ANOVA was executed for each kinematic variable to determine whether the agreement was different between groups. The test-retest reliability of each kinematic variable was evaluated for both systems using ICCs and Bland-Altman analysis. The test-retest reliability was determined between the first two trials in which the same lead foot crossed the obstacle. A group (AMD subjects, older controls, younger controls) by system (2-D, 3-D) mixed ANOVA was performed for each kinematic variable to determine whether the test-retest reliability was different between groups and systems. Significant *F* values from the ANOVA’s were subjected to a Bonferroni post hoc pairwise comparison to determine the means that were different. The level of significance (α) was set at *p* < 0.05. Effect sizes were calculated using Cohen’s *d*.

## Data Availability

The data that support the findings of this study are available from the corresponding author, T.Z., upon reasonable request.
